# A distributed computing model for big data anonymization in the networks

**DOI:** 10.1371/journal.pone.0285212

**Published:** 2023-04-28

**Authors:** Farough Ashkouti, Keyhan Khamforoosh

**Affiliations:** 1 Department of Computer Engineering, Mahabad Branch, Islamic Azad University, Mahabad, Iran; 2 Department of Computer Engineering, Sanandaj Branch, Islamic Azad University, Sanandaj, Iran; Kocaeli Üniversitesi: Kocaeli Universitesi, TURKEY

## Abstract

Recently big data and its applications had sharp growth in various fields such as IoT, bioinformatics, eCommerce, and social media. The huge volume of data incurred enormous challenges to the architecture, infrastructure, and computing capacity of IT systems. Therefore, the compelling need of the scientific and industrial community is large-scale and robust computing systems. Since one of the characteristics of big data is value, data should be published for analysts to extract useful patterns from them. However, data publishing may lead to the disclosure of individuals’ private information. Among the modern parallel computing platforms, Apache Spark is a fast and in-memory computing framework for large-scale data processing that provides high scalability by introducing the resilient distributed dataset (RDDs). In terms of performance, Due to in-memory computations, it is 100 times faster than Hadoop. Therefore, Apache Spark is one of the essential frameworks to implement distributed methods for privacy-preserving in big data publishing (PPBDP). This paper uses the RDD programming of Apache Spark to propose an efficient parallel implementation of a new computing model for big data anonymization. This computing model has three-phase of in-memory computations to address the runtime, scalability, and performance of large-scale data anonymization. The model supports partition-based data clustering algorithms to preserve the λ-diversity privacy model by using *transformation* and *actions* on RDDs. Therefore, the authors have investigated Spark-based implementation for preserving the λ-diversity privacy model by two designed City block and Pearson distance functions. The results of the paper provide a comprehensive guideline allowing the researchers to apply Apache Spark in their own researches.

## 1. Introduction

Organizations such as hospitals, universities, telecommunication centers, and social networking sites publish their data on the web for research society or deliver it to other organizations to analyze and extract its hidden patterns [1]. But sharing, publishing, and analyzing original datasets may breach individuals’ privacy. Therefore, modern privacy-preserving methods should be developed for data publishing and analysis to obstruct the disclosure of individuals’ sensitive information [2–4]. In general, methods such as access restriction, encryption, anonymization, and noise-based methods are used for privacy preservation [3]. Anonymization, which is the surveyed method in the paper, is the commonest method of privacy-preserving in data publishing (PPDP) to provide the different privacy models such as k-anonymity [5], λ-diversity [6], and t-closeness [7]. Although many advances have been achieved in the data privacy field, there are still significant challenges in this realm. One of these challenges is the growth of data and the big data matter [8–10]. Some of the most important characteristics of big data are Volume, Velocity, Variety, Veracity, Value, Visualization, Vulnerability, and Virality. Volume is, how much data is available? The dataset has to be large in scale, like Gigabyte, Terabyte, Petabyte, etc. Velocity is, how fast are new data generated? The speed of data generation in social networks, IoT, healthcare applications, search queries, and other companies are rising every second. Variety is, how diverse are the data? Data may be in many types, likes database, XML, text, image, video, audio, log files, click data, etc. Veracity is, how much is the quality? Data have to be accurate, correct and trusted. Value is, how much value is the data? Having big data is useful if it leads to valuable information. Visualization is, how challenging is data to visualize? Due to limitations in traditional technologies, representing big data have faced new challenges. Therefore, new methods are required for representing big data like millions of data points. Vulnerability is how much information can be found based on other information? Big data has brought new *security* and *privacy* [11] challenges. Unfortunately, there have been many big data breaches. These breaches may disclose user account information, identity, etc. Virality is how quickly information propagates through social networks? For sharing information across a network, the main factor is time [12–15].

Given the mentioned above features for big data, privacy-preserving in big data publishing (PPDBP) requires new algorithms, architectures, models, and platforms for big data anonymizaion [16,17]. Recently, Apache has released some platforms such as, Hadoop and Spark [18] for big data management. Apache Spark is a fast, open-source, massively parallel, scalable, and general engine for large-scale data processing. Hadoop processes data on disk, whereas Apache Spark [19] uses in-memory multi-staged computation in form of RDDs (Resilient Distributed Datasets), which causes extremely fast distributed data processing. In-memory data processing is a key feature that enables Apache Spark to process the data 100 times faster than Hadoop. Therefore, Apache Spark is an advantageous framework to address the runtime, scalability, and performance of large-scale data anonymization. Apache Spark distributes intensive data processing on computational worker nodes and improves processing performance by parallelizing anonymization computations [20].

**Fig 1:**
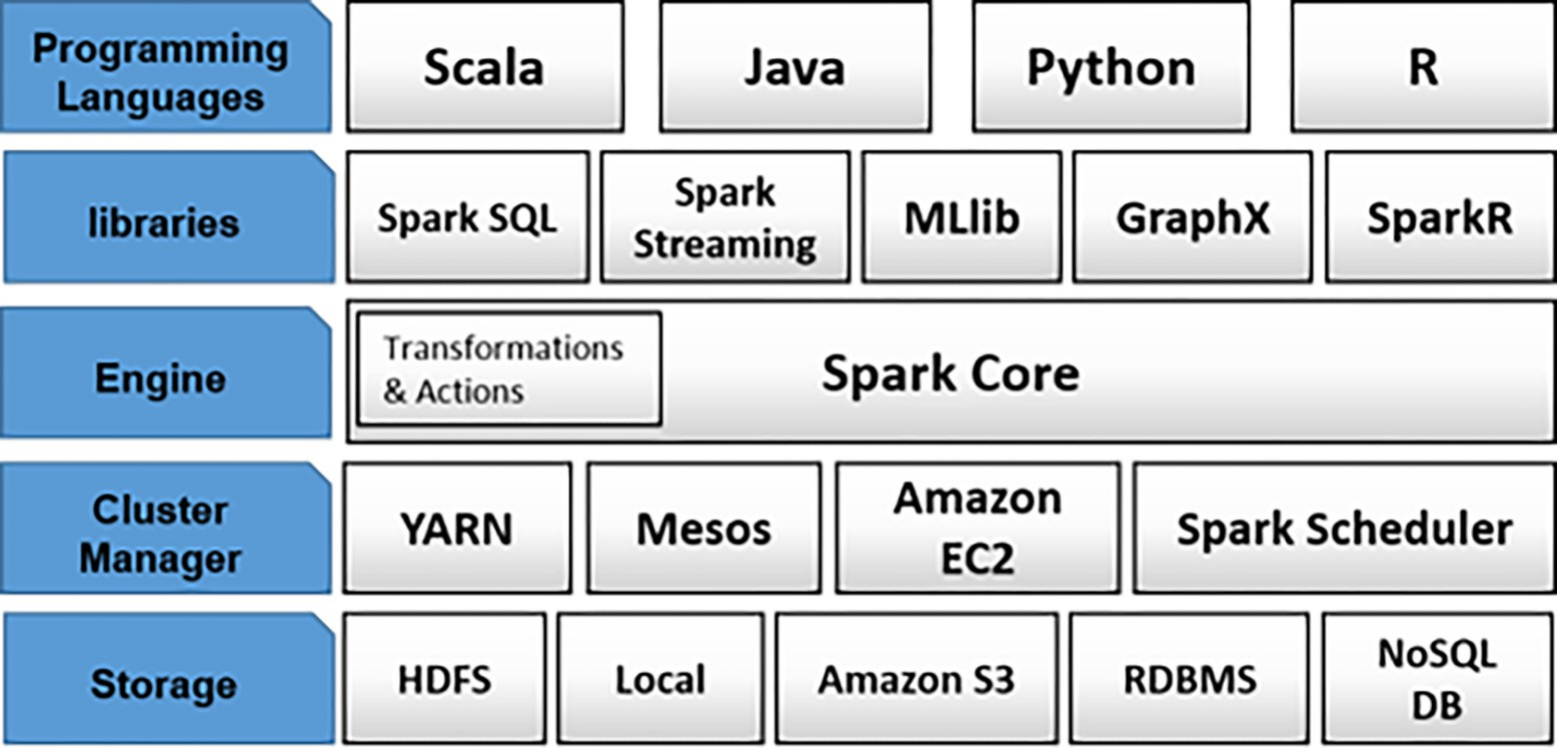
Spark ecosystem [22].

The Apache Spark ecosystem is illustrated in Fig 1. The layers of the ecosystem from top to bottom include programming languages, libraries, engine of spark, cluster manager, and storage. The programming languages layer provides different languages to develop distributed applications. Library Layer has several powerful libraries to work with SQL, machine learning, stream, and graph data. Spark Core is the engine of Spark and is responsible for distributing and writing large-scale data on clusters, executing operations on clusters, memory management, etc. In the cluster manager layer, running the Spark framework requires connecting to one cluster manager, and in the storage layer, it requires a distributed file system or database for storing the big data [21].

The resilient distributed dataset (RDD) is the Spark core abstraction for operating on datasets. Each RDD is broken into multiple partitions and is distributed on the cluster nodes. Two types of operations are performed on RDDs: 1. *Transformation*, which is performed on an RDD and returns another RDD (like map() and filter()), and 2. *Action*, which is performed on an RDD, and returns a distinct value (like reduce() and count()). A Spark application includes transformations and actions. When a Spark application calls an action (like collect()) on an RDD, a job is created. The created job is broken into one or multiple stages, each being a transformation. Then, the stages are decomposed into tasks. Tasks are units of execution in Apache Spark. The driver’s scheduler distributes the tasks among executors at the worker nodes to run them in parallel [18,22].

**Fig 2:**
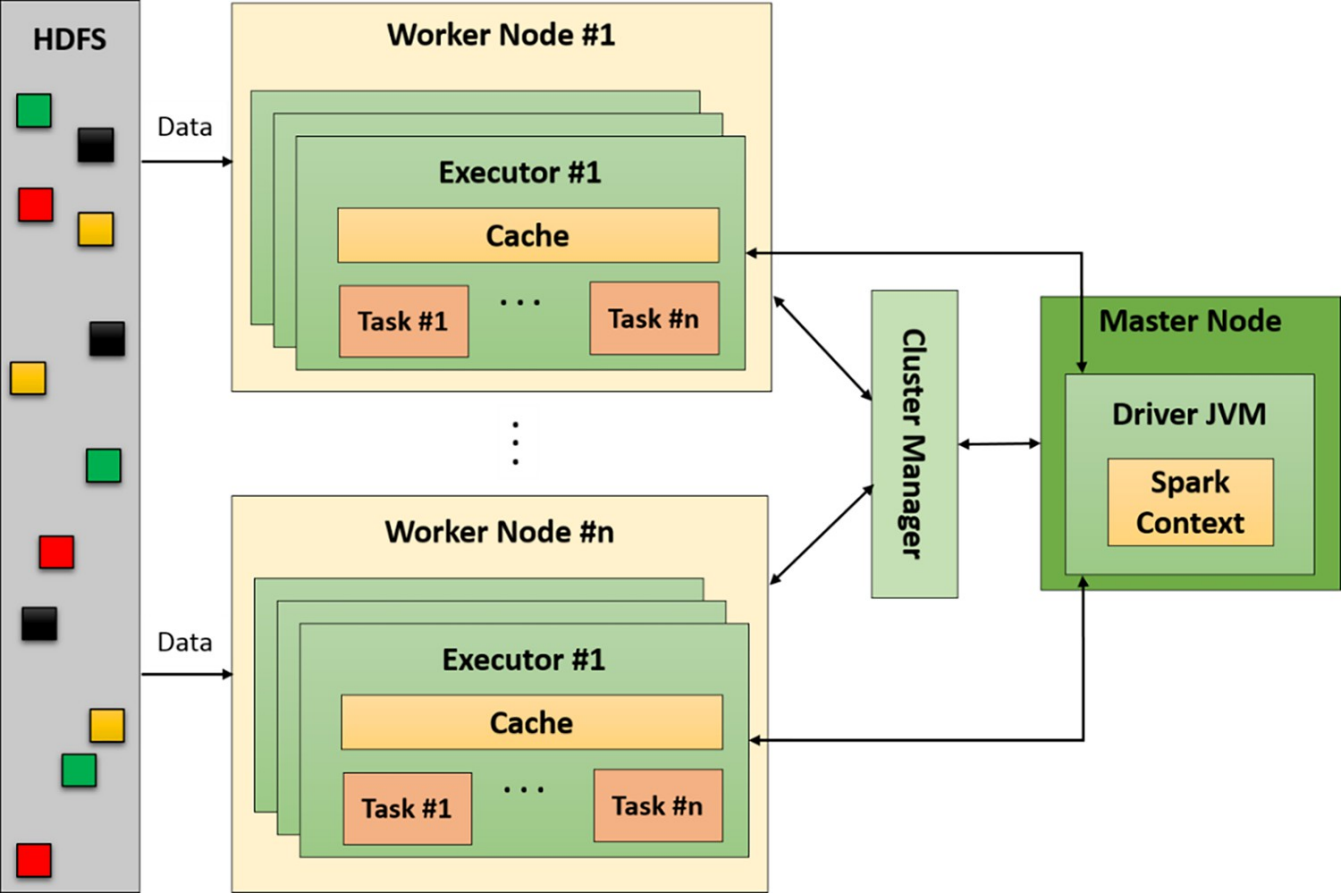
Spark cluster architecture [19,21].

There is an overall view of the Spark cluster architecture in Fig 2. Once an application is launched on a cluster, the SparkContext connects to the cluster manager. Then, the cluster manager finds executors on worker nodes, allocates them to the application, and reads the data from HDFS. Next, SparkContext sends the application code (Python or JAR files) to the executors, on which all the tasks run [19,23].

As mentioned above, Spark is an in-memory iterative computing framework for large-scale data processing. It is the state-of-the-art framework for implementing the iterative operations of data anonymization on large-scale datasets. Three Contributions in the present paper are:

- An efficient three-phase computing model has been suggested for big data anonymization using the RDDs programming in Spark. Our model supports the iterative operations of partition-based data clustering algorithms such as k-means, k-medoid, etc. All of the three-phase of the model has been implemented by Transformations and Actions on the RDDs.
- This computing model is specialized for preserving the λ-diversity privacy model and is flexible to preserve the other privacy models such as t-closeness. Two distance functions have been designed using city block and Pearson distances for satisfying the λ-diversity privacy model requirements. Therefore, the challenges related to scalability, runtime, and even information loss are addressed.
- This model provides general knowledge for researchers and engineers to use the Apache Spark framework in their fields.

In the remainder of the paper, the following sections are included. Section 2 introduces privacy-preserving models in data publishing and a preliminary of data anonymization operators. Section 3 reviews applications of Apache Spark in different fields, and then surveys the related works of distributed methods for big data anonymization. In section 4, the proposed computing model is described. Section 4 evaluates the proposed computing model, and in section 5 the conclusions are presented.

## 2. Preliminiaries of data anonymization

In the k-anonymity privacy model, data samples are grouped in at least k members and at most 2k-1 member groups. In the anonymization process, the value of all quasi-identifier attributes in each group is modified to the same value. Thus, the data sample or the individuals’ identity becomes indistinguishable among the data samples of the group [5]. There is a sample medical dataset in Table 1, which contains the individuals’ SIN identifier, and publishing it causes disclosing individuals’ sensitive information. At first, by an anonymization algorithm, SIN is removed from the dataset. Then, the groups are made, and the values of three other attributes (quasi-identifiers) have been generalized to equal values. Therefore, in all groups, the data samples are not distinguishable from each other and the 4-anonymous dataset is published according to Table 2. Now, if a data attacker has background knowledge about somebody, he is incapable of re-identify the associated data record, because the probability of re-identifying any data sample is not more than 1/k. Generally speaking, the k-anonymity model is resistant to record-linkage attacks [13].

**Table 1:** A sample medical dataset.

SIN	Gender	Age	Country	Sickness
76890982	M	20	USA	Flu
21368903	M	25	Mexico	HIV
34565908	M	25	Canada	Covid-19
89645660	M	29	USA	Flu
21369877	M	39	Italy	HIV
98003456	M	32	USA	depression
67567810	M	30	France	depression
90234556	M	37	Canada	Flu
18904409	F	40	Germany	Covid-19
23387390	F	43	U.K	Covid-19
11789034	F	45	France	Covid-19
98034556	F	49	U.K	Covid-19

**Table 2:** Medical dataset in 4-anonymous model.

Gender	Age	Country	Sickness
M	20–29	America	Flu
M	20–29	America	HIV
M	20–29	America	Covid-19
M	20–29	America	Flu
M	30–39	*	HIV
M	30–39	*	Depression
M	30–39	*	Depression
M	30–39	*	Flu
F	40–49	Europe	Covid-19
F	40–49	Europe	Covid-19
F	40–49	Europe	Covid-19
F	40–49	Europe	Covid-19

The k-anonymity privacy model can not preserve the sensitive attributes (like sickness in Table 1) against other attacks. For example, assume Sophia has been recently hospitalized for sickness, and a data attacher wants to discover her sickness, which is highly private to her. Therefore, data attacker lookups in Table 2. Since Sophia is a 43-year-old woman from Germany and the data attacker has this knowledge, so Sophia’s information is known to the attacker, then he finds out that her record is in the third group. Because all the records of the third group have Covid-19, therefore attacker realizes that Sophia also suffers from Covid-19. Thus, the attacker doesn’t require identifying Sophia’s exact record in the third group.

In this example, private information has been disclosed due to the uniformity and lack of diversity in the sensitive attribute. Thus, the k-anonymous datasets (like Table 2) are exposed to the attribute linkage attacks [8,10].

The λ-diversity privacy model provides more data protection than k-anonymity. In this model, not only the groups must have at least k data samples but also all groups must have at least λ different values for the sensitive attribute. Therefore, the data attacker is incapable to detect the individuals’ records by using the value of the sensitive attribute. Thus, this model preserves the published data against attribute linkage attacks. By an anonymization algorithm, the dataset has been generalized to the 3-diversity privacy model and published in Table 3. The data values of Table 3 are more general than Table 2 because there must be at least 3 distinct values for sickness in all groups. Now, the attacker can not detect Sophia’s record in the second group, and identifying her sickness has been more complicated, and the sickness is not discoverable [6].

**Table 3:** Medical dataset in 3-diversity model.

Gender	Age	Country	Sickness
M	20–39	*	Flu
M	20–39	*	HIV
M	20–39	*	Covid-19
M	20–39	*	Flu
M	20–39	*	HIV
M	20–39	*	depression
*	30–49	*	depression
*	30–49	*	Flu
*	30–49	*	Covid-19
*	30–49	*	Covid-19
*	30–49	*	Covid-19
*	30–49	*	Covid-19

**Fig 3:**
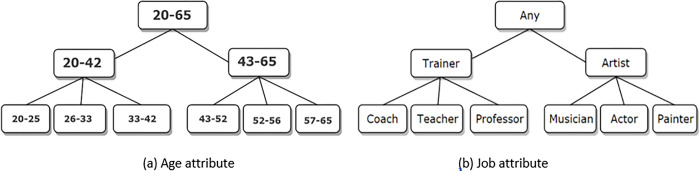
Generalization tree for age and job attributes.

Data anonymization is generally carried out as a generalization, suppression, permutation, anatomization, or perturbation operator to provide one of the privacy models such as k-anonymity [5], λ-diversity [6], or t-closeness [7] and the idea behind all of these approaches is data modification. Generalization and suppression replace QID values with more general values. Permutation and anatomization de-associate the relationship between sensitive attributes and quasi-identifiers by shuffling and grouping data values in an equivalence class. Perturbation modifies the data values by adding noise, swapping, aggregating, etc. Generalization is the commonest and most powerful data anonymization approach and a lot of algorithms have been designed based on it [24]. In this paper, the generalization operator for anonymization is surveyed. Generalization uses a hierarchical tree of values for attributes that from bottom to up the values become more general. The generalization tree for numeric and categorical attributes is illustrated in Fig 3.

Generalization has five different schemas and briefly has been described in the following [24,25].

- **Full Domain Generalization:** Generalization occurs in the whole of the tree. All values of an attribute are replaced by a value of a similar level in the hierarchy tree. That is, all leaf nodes are generalized to the same level of non-leaf nodes. For example, in Fig 3, if Coach, Teacher, and Professor are replaced by Trainer, then it must be that Artist replaced Musician, Actor, and Painter.
- **Subtree Generalization:** In this schema, generalization occurs for values of some nodes, and values of other nodes can be ungeneralized. That is in non-leaf nodes of the hierarchy tree, none of the child values are generalized, or all of them are generalized. For example, in Fig 3, if Coach was generalized to Trainer then Teacher and Professor must be generalized to Trainer. In another part of the tree, Musician, Actor, and Painter should remain ungeneralized, or all of them should be generalized to Artist.
- **Sibling Generalization:** This is similar to the subtree generalization schema. But if the value of a leaf node has been generalized, then it’s not required for the value of its sibling nodes to be generalized. For example, in Fig 3, if Teacher generalized to Trainer, then Professor can be ungeneralized.
- **Cell Generalization:** This schema is called local recoding. Occurrences of a data value in some records of a dataset can be generalized, and in others can be ungeneralized. For example, in Fig 3, Painter in some records is generalized to Artist and in other records remains as Painter.
- **Multidimensional Generalization:** The generalization methods mentioned above are single-dimensional, and there is a separate generalization function for each QID attribute. But in multi-dimensional generalization schema (like Mondrian algorithm), there is a single function for all QID attributes. This function generalizes values of all QID attributes for all records in an equivalence class to the same values.

## 3. Related works

Recently, distributed implementation of big data anonymization techniques has become one of the main challenges in big data community research. Distributed methods and parallel computations address the corresponding challenges toward the scalability, runtime, and performance of algorithms [8,9,13]. The following is a brief overview of Apache Spark applications in different fields. Then, the distributed methods of big data anonymization to preserve the k-anonymity and λ-diversity privacy models by MapReduce and Apache Spark are surveyed.

### 3.1 Apache Spark applications

Among the state-of-the-art parallel computing frameworks, Apache Spark is a fast, general-purpose, and in-memory computing framework for big data processing that provides high speed and scalability in computations. Recently, Spark has been one of the most popular parallel processing frameworks. This framework has been applied in different fields such as bioinformatics [26], social networks [27], information security [28], and the Internet of Things(IoT) [29]. In the following, some applications of Spark are reviewed.

Runxin et al. [26] used apache spark as a parallel computing framework in the analysis of genomic data. They proposed a distributed model by RDD programming to use Spark-based applications in NGS and other biological domains. Samar et al. [27] used Spark as a large-scale sentiment data classification for analysis of online Reviews. They applied SVM, Naïve Bayes, and logistic regression as data classifiers from the machine learning library (MLlib) of Spark. Gupta et al. [28] proposed a framework for a cybersecurity intrusion detection system. They used Apache Spark as a big data processing tool for processing large size of network traffic data. This framework used correlation-based feature selection and Chi-squared feature selection and five classification-based intrusion detection methods, namely, Logistic regression, Support vector machines, Random forest, Gradient Boosted Decision trees, and Naive Bayes for analysis of the network traffic. Panigrahi et al. [30] proposed a hybrid distributed algorithm for a collaborative filtering recommender engine by parallel implementation of Spark. To overwhelm the sparsity and scalability challenges, it used dimensionality reduction techniques like alternating least square and clustering techniques like k-means. This method also diminishes the cold start problem of collaborative filtering by correlating the users to products through features (tags). Manar et al. [31] have proposed a Comprehensive Storing System for COVID-19 data using Apache Spark (CSS-COVID) to address the problem caused by increasing the number of COVID-19 daily. CSS-COVID consists of three stages, namely, inserting and indexing, storing, and querying stage. In the inserting stage, data is divided into subsets and then indexed each subset separately. The storing stage uses a set of storing nodes to store data while the querying stage is responsible for handling the querying processes. Using RDDs programming and Spark libraries, CSS-COVID handles and manages large-scale data of the coronavirus disease injured which increase daily.

### 3.2. Distributed anonymization methods

Distributed methods of large-scale data anonymization have been developed to address the challenges related to scalability and runtime [32,33]. Most of these methods have been presented using the Hadoop MapReduce and some of them are designed on Apache Spark.

Ashkouti et al. [34] proposed a Distributed Improved Mondrian (DI-Mondrian), to preserve the λ-diversity privacy model using RDDs programming in Apache Spark. DI-Mondrian uses information gain and coefficient of variation as selecting the cut-dimension and has a new dynamic method for selecting the cut-points. The result of experiments proved the outstanding performance in information loss, data utility, and runtime. Bazi et al. [35] proposed an RDD-based subtree generalization implementation strategy for Apache Spark. The method reduces the complexity of operations and improves performance by the use of effective partition, improved memory, and cache management. They showed the proposed approach offers high scalability and performance through a better selection of subtree generalization processes and data partitioning. Canbay et al. [20] introduced Apache Spark as a distributed processing framework for privacy-preserving in big data. No algorithm has been proposed in the paper, but a computing model has been introduced for big data anonymization within the Spark cluster. Zakerzadeh et al. [36] proposed two scalable distributed algorithms based on the MapReduce platform for big data publishing. They have presented two baseline and improved algorithms, which run over the Mapper, Combiner, and Reducer computational nodes. These algorithms preserve the k-anonymity and λ-diversity privacy models. Zhang et al [37]. have proposed a scalable LSH-based clustering algorithm for big data anonymization on MapReduce. They have designed a new metric for measuring the distance between data records and constructed the k-member clusters to preserve k-anonymity privacy model. In another research, the same authors [38] have proposed a scalable two-phase big data anonymization algorithm over MapReduce for cloud-based platforms. In each phase, a group of MapReduce jobs is designed, which carry out the top-down specialization operations in a scalable fashion to preserve the k-anonymity privacy model. In another study, the same authors [39] designed a distributed method over MapReduce which uses two-step clustering. A metric has been designed using the quasi-identifier and sensitive attributes that calculate the distances between the data records. The data are then grouped into at least k-members clusters using the above distances. In another research, the same authors [40] have proposed a hybrid method based on MapReduce to address the scalability of big data sub-tree anonymization in the cloud environment. Since top-down specialization (TDS) and bottom-up generalization (BUG) provide low scalability, the designed method, using a mixture of these algorithms, has implemented two scalable MapReduce-based algorithms, to gain high scalability in sub-tree anonymization. Nayahi et al. [41] proposed a novel scalable clustering algorithm over Hadoop for big data anonymization. After the anonymization process, the data are distributed over the Hadoop distributed file system (HDFS) and are resistant against probabilistic inference attacks, similarity attacks, and other ordinary attacks. Al-Zobbi et al. [42], to address the challenges related to scalability and performance in PPBDP, have proposed a framework for big data anonymization. In their framework, Pig Latin and UFD programming have been applied, which increases the speed of the MapReduce computations. This framework performs horizontal data fragmentation based on the class attribute value (not randomly), which decreases information loss. Jain et al. [43] have designed a new layer between HDFS and MapReduce that improves data sharing for data analysis. The model (SMR model) is scalable, performs its operations as the Map and Reduce jobs, and is intended to establish a better trade-off between privacy and data utility.

As introduced in this section, most of the distributed algorithms in the literature have been developed using the MapReduce platform to satisfy the k-anonymity privacy model. But, due to the excellence of Spark in scalability and performance, recent trends to design novel methods in Spark have been raised. Therefore, the main goal of this paper is to propose a computing model in the Apache Spark framework for big data anonymization, which addresses the scalability and performance of the former platforms. The proposed computing model uses partition-based data clustering algorithms to preserve the λ-diversity privacy model that has a higher level of data protection than k-anonymity. The RDDs programming with Transformation and Actions in the Apache Spark framework is used for parallelizing in-memory operations. The focus of the proposed computing model is to improve the scalability and performance of big data anonymization techniques and then provide general knowledge for the researchers and engineers to use the Apache Spark framework in their fields.

## 4. Proposed method

This section first presents the use of partition-based clustering methods in a hierarchical way to form sub-clusters that preserve the requirement of privacy models. Next, a three-phase computing model is proposed for anonymizing big data on the Spark framework. This model is implemented by RDDs programming to preserve the λ-diversity privacy model.

### 4.1 Data clustering schema by partioning algorithms on Apache Spark

Data clustering methods place the data objects into groups, or clusters, such that objects in a given cluster tend to be similar to each other in some sense, and objects in different clusters tend to be dissimilar. These methods have various applications such as outlier detection [44], image segmentation [45], information retrieval [46], social networks [47], and System recommanders [48]. In the data privacy field, these techniques are useful to group data objects into at least k-member clusters that preserve privacy models such as λ-diversity. These methods are divided into several categories such as partitioning algorithms, hierarchical algorithms, density-based algorithms, and grid-Based algorithms [46]. This paper proposes a distributed three-phase computing model based on partitioning algorithms (such as k-means and k-medoid) to preserve the λ-diversity privacy model by apache spark.

**Fig 4:**
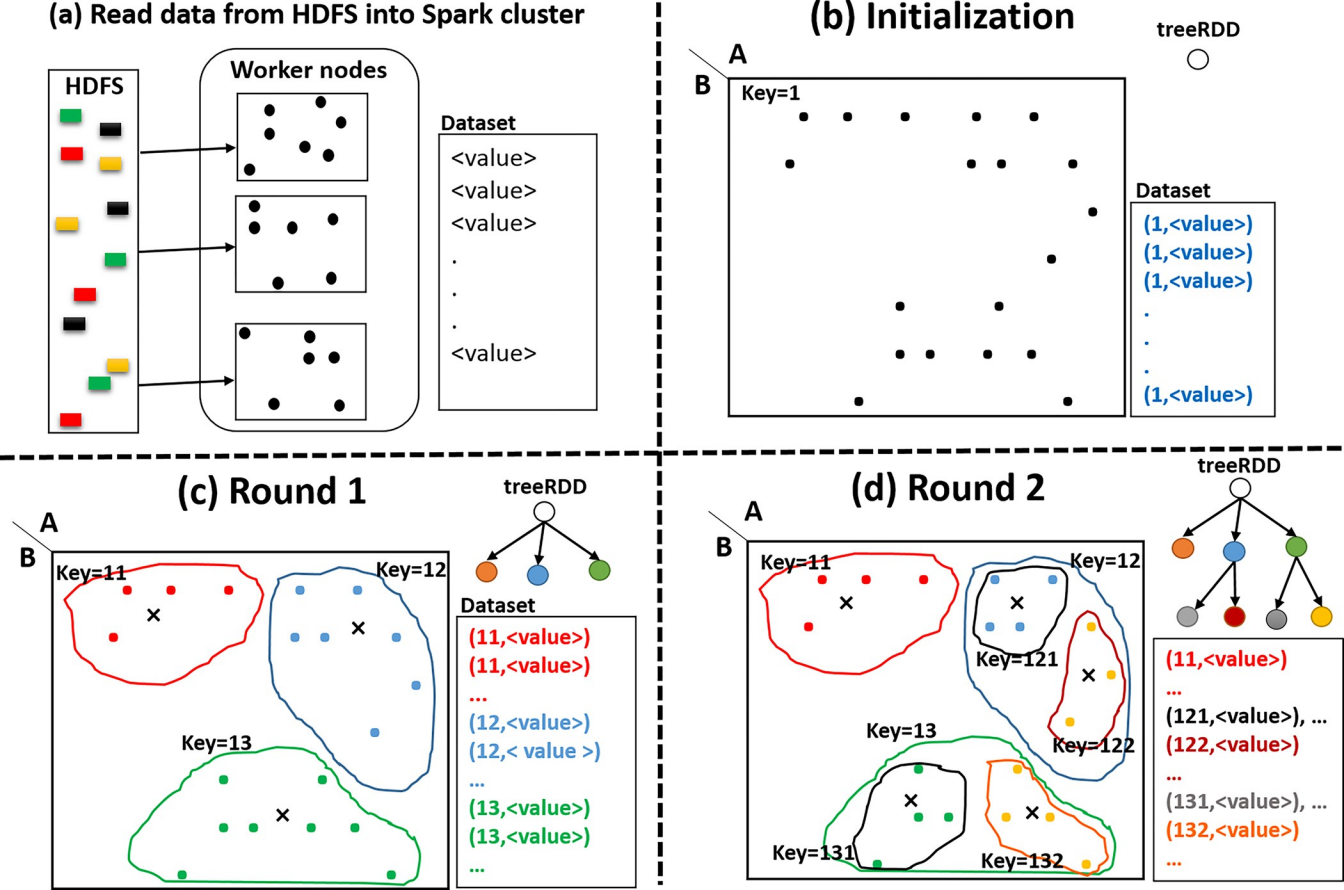
Schema of hierarchical data clustering in Spark framework: (a) reading dataset from HDFS into worker nodes. (b) Assigning a unique key to all recrods. (c) The first round of data clustering and forming sub-clusters. (d) The second round of data clustering and forming smaller sub-clusters.

Since the k-anonymity privacy model is the basis of the λ-diversity privacy model, Fig 4. uses the k-anonymity model to introduce the concept of hierarchical data clustering to form at least k-member clusters in the Apache Spark framework. It illustrates the hierarchical data clustering steps on 19 data samples to preserve the 3-anonymous privacy model. As shown in Fig 4(A), at first all data records are read and distributed into the memory of the worker nodes. In the proposed model, the <*key*, *value*> structure is used to show each data record. This structure determines the belonging of each data record to its relevant cluster. In this structure, *key* is a unique identifier assigned to each cluster, and *value* is the data values of the record. Fig 4(B), illustrates the initialization step. Since data clustering has not been initiated, then all data records belong to the largest cluster. Hence, *key* of the all data records is set to 1. On the other hand, to maintain the hierarchical structure of data clustering and the cluster centers at each execution round, a generic tree(treeRDD) with the <*key*, *value*> structure is used. In this structure, *key* is the key of the cluster and *value* is the selected cluster centers (seeds). At this stage, the treeRDD has only the root node, which is *key* = 1 and *value* = null (<1, null>). Therefore, data records with the same *key* belong to the same cluster, and if the record key is equal to the key of each leaf node in the treeRDD, this record is placed in that cluster.

**Algorithm 1. Data anonymization in Spark**

**Input:** dataset

**Output:** anonymizedRDD

1. dataRDD = read dataset from hdfs and distribute them among worker nodes of spark cluster

2. pairdataRDD = establishing <1,value> pairs by data records

3. treeRDD = establishing root node of tree with <1,null> structure

4. initialization k, λ parameters and broadcast them on spark cluster

5. while (is there any cluster can split more?)

6. {

7.     seedRDD = selecting k’ seeds as cluster centers by proposed dist() function

8.     cluster_flag = 0

9.     while (cluster_flag = = 0)

10.      {

11.             i = 0

12.         ϴ = iterations of kmeans

13.             //data will be clustered by an algorithm such as k-means

14.             While (i < = ϴ)

15.             {

16.                 foreach (record in dataset)

17.                     assign each record to neareset seed of seedRDD by proposed dist() function

18.                 new_key[] = updating seeds by kmeans algorithms (averaging of data values)

19.                 pairRDD = establishing new sub-clusters by updating pairRDD as <new_key,value>

20.             }

21.             seedRDD = updating seedRDD by new_key[]

22.             // checking the λ-diversity conditions

23.           if (some sub-clusters don’t meet λ-diversity condition)

24.           {

25.                 seedTemp = seed of clusters don’t meet λ-diversity condition

26.                 seedRDD = seedRDD–seedTemp

27.                 // now data will be re-clusterd by the remaining seeds of seedRDD in the next attempt

28.           }

29.           else

30.             cluster_flag = 1 //accepting that all sub-clusters are l-diverse

31.       } //end of establishing sub-clusters

32.       treeRDD = updating value part of tree nodes by adding final seeds

33.       treeRDD = adding new nodes to tree with unique key

34.     } //end of an execution round

35.     anonymizedRDD = generalizing the dataset by treeRDD

Fig 4(C) shows the first execution round of hierarchical data clustering. In this example, three cluster centers (seeds) are selected, and three sub-clusters are formed using one of the partition-based clustering algorithms. Therefore, three nodes <11,s1>, <12,s2>, and <13,s3> are appended to the treeRDD. The data records are now placed in one of three new sub-clusters, and *key* of all data records is updated with the corresponding cluster *key*. After data clustering, all data records must be placed in at least 3-member sub-clusters, and if a cluster has less than three records, the seed of that cluster must be removed and its data records appended to the nearest cluster. In Fig 4(C) the data records have been updated as <11,value>, <12,value>, and <13,value> and have shown in red, blue, and green, respectively. Fig 4(D) shows the second execution round, where the further clustering operations are continued on the clusters with key = 12 and key = 13. In this round, new nodes are appended to the treeRDD by forming new sub-clusters. Because all the formed sub-clusters are 3-anonymous and further divisions are not allowed, then the operations are terminated. More details of this operation are provided in Algorithm 1.

### 4.2 The proposed computing model

**Fig 5:**
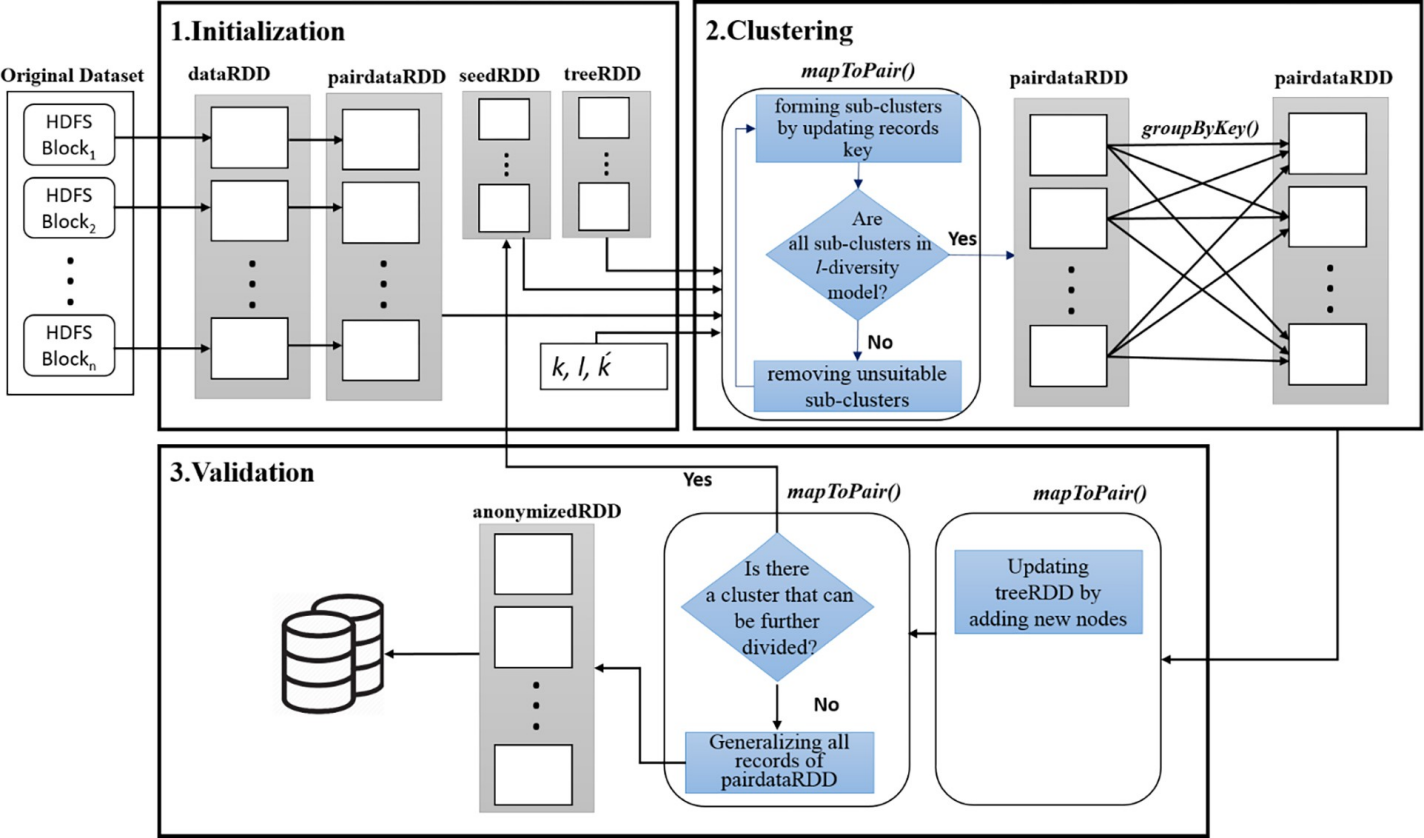
Main steps of the proposed three-phase computing model.

In this section, we present a description of our proposed computing model for Spark-based anonymization. Our computing model consists of three phases: 1. Initialization, 2. Clustering, and 3. Validation. As shown in Fig 4, the proposed model uses the hierarchical implementation of partition-based clustering algorithms to preserve the λ-diversity privacy model. Fig 5 illustrates the in-memory operations on the RDDs in the proposed computing model. In each step indicated with a rectangle, an RDD is established. For RDDs establishment and implementation of our computing model, Apache Spark *Transformations* and *Actions* are used. In the following, the details of each phase are described.

#### 4.2.1 Phase 1: Initialization

One of the advantages of the Spark framework is the parallelization of in-memory computations in the form of RDDs to speed up the computations. In the initialization phase of the proposed computing model, the required RDDs and broadcast variables of the model are established and distributed as follows.

- **dataRDD.** First, the entire dataset is read from the HDFS and placed in the dataRDD. The textFile() method of the Spark Context class is used to establish and distribute the datasetRDD on the executor of worker nodes.
- **pairdataRDD.** As illustrated in Fig 4(B), the proposed computing model uses the <*key*, *value*> structure for the operations, and in this phase using the dataRDD a new RDD is established with the <*key*, *value*> structure, namely, pairdataRDD. In Java, the mapToPair() Transformation (in python map()) is used to add *key* = 1 to all data records and establish pairdataRDD.
- **seedRDD.** In each execution round, ḱ records are selected as the cluster centers to form sub-clusters. The seed selection can be random (like ḱ-means) or be more intelligently (like ḱ-means++). In the next section, the designed city block and pearson distance functions based on the requirements of the λ-diversity privacy model are introduced for selecting the cluster centers. The selected seeds are placed in seedRDD and established by a mapToPair() transformation.
- **treeRDD.** As illustrated in Fig 4, in all execution rounds the treeRDD is used to maintain the hierarchical structure of data clustering. The treeRDD has the <*key*, *value*> structure and is established by a mapToPair() transformation.
- **k, λ, and ḱ variables.** Some parameters are required for the anonymization process and are broadcasted on the executor of worker nodes. Therefore, parameters such as k in k-anonymity, λ in λ-diversity, and ḱ (number of seeds) are defined as broadcast variables. In the instruction_set 1, k and λ are initialized with 120 and 4 respectively then, they are broadcasted on the Spark cluster.

The introduced RDDs and variables are established and distributed in the Spark computational cluster as instruction_set 1. The required operations of the mapToPair() transformations are accomplished by a passed function to it that has been indicated by three dots.

**Table:** 

Instruction_set *1*. Initialization Phase
Input: Original Dataset Output: Required RDDs and Variables
1. JavaRDD<String> dataRDD = sc.textFile(hdfs://…/dataset.txt); 2. JavaPairRDD<Key, Value> pairdataRDD = dataRDD.mapToPair(…); 3. JavaPairRDD<Key, Value> seedRDD = pairdataRDD.mapToPair(…); 4. JavaPairRDD<Key, Value> treeRDD = pairdataRDD.mapToPair(…); 5. Broadcast<Integer> k = sc.broadcast(120); 6. Broadcast<Integer> λ = sc.broadcast(4);

#### 4.2.2 Phase 2: Clustering

In this phase, the distributed data records on the worker nodes are grouped in λ-diverse clusters. In the proposed computing model, all the partition-based clustering algorithms (such as k-means, k-medoids, k-medians, k-modes) or different versions of them (such as k-means++, kernel k-means, CLARA, CLARANS) are usable. For forming sub-clusters, the clustering algorithm uses the cluster centers of seedRDD and data records of pairdataRDD simultaneously. In Apache Spark, for simultaneously accessing two RDD must join them. Therefore, *seedRDD* and *pairdataRDD* are joined by *join()* transformation. The general operations of the clustering phase are as instruction_set 2.

**Table:** 

Instruction_set *2*. clustering Phase
Input: seedRDD, pairdataRDD, broadcasted variables (k,l, and ḱ) Output: updated pairdataRDD
1. temp = pairdataRDD.join(seedRDD); 2. pairdataRDD = temp.mapToPair(…); 3. pairdataRDD = pairdataRDD.groupByKey(…);

The operations of mapToPair() transformation of this phase are shown in Fig 5. Initially, a clustering algorithm uses an λ-diversity-based distance function (such as the Euclidean, City block, Cosine, Pearson, or other distances) to update the cluster centers iteratively and form final sub-clusters. As shown in Fig 4(C), the structure of records is the <*key*, *value*> and new sub-clusters are created by updating the record’s *key* and assigning a new *key* to them. Hence, records with the same *key* are placed in the same cluster. Next, all sub-clusters are examined to determine whether the requirements of the λ-diversity privacy model are preserved or not. If some clusters do not preserve the λ-diversity requirements, the centers of those clusters are removed from the seedRDD, and then the clustering operation is performed again in another attempt. After performing the operations of the mapToPair() transformation, the groupByKey() transformation is executed on the pairdataRDD. This transformation shuffles the <*key*, *value*> pairs so that pairs with the same *key* are placed on the same executor of worker nodes. Therefore, the pairdataRDD is prepared on the Apache Spark computing cluster for the next execution round.

According to the λ-diversity model requirements, each cluster must have at least k records and there must be λ distinct values for the sensitive attribute [6]. Therefore, in all execution rounds to form more cohesive clusters, firstly, records must be clustered in at least k-member clusters in which values of the quasi-identifier attributes are close to each other. Secondly, the values of the sensitive attributes must be far. In this paper, two λ-diversity-based distance functions are designed, namely, 1. City block distance function [49], and 2. Pearson distance function [50]. In addition to data clustering using the designed distance functions, they are also usable for seed selection. The choice of a distance measure depends on the nature of the data, and the most important consideration is the type of the features of the objects. Since our method has been proposed for numerical datasets the city block distance is used which is efficient in computing the real distance between samples. City block takes into account the paths that realistically could be taken within the values of those attributes. On the other hand, the person distance metrics can support calculating the distance between data samples with at least two sensitive attributes that occur in some cases.

At first, the data values of all records are normalized using one of the normalization methods such as Min-Max, Z-Score, or decimal scaling [51]. Then, the distance of two numeric values is calculated using Eq (1). This distance is the absolute value of the difference between the numeric values *v*_1_ and *v*_2_.

$$d(v_{1},v_{2})=|v_{2}-v_{1}|$$ (1)

To calculate the similarity of two sensitive values, the normalized distance of them is subtracted from 1. Then, the similarity of numeric sensitive values *sv*_1_ and *sv*_2_ is calculated as Eq (2).

$$d{}_{sim}(sv_{1},sv_{2})=1-d({sv}_{1},{sv}_{2})$$ (2)

*City block distance function*. Eq (3) is the designed city block distance function based on the λ-diversity requirements. This function calculates the city block distance between numeric records *r*_*a*_and *r*_*b*_ that have quasi-identifier (QID) and sensitive attributes (SA).

$$dist(r_{a},r_{b})=\sum_{i=1}^{\#qid}\left(w_{i}^{qid}.d_{i}^{qid}(v_{1},v_{2})\right)+\sum_{j=1}^{\#SA}{(w}_{j}^{SA}.{d{}_{sim}}_{j}^{SA}(sv_{1},sv_{2}))$$ (3)

In Eq (3), $w_{i}^{qid}$ and $w_{j}^{SA}$ are the weights of the quasi-identifier and sensitive attributes. The weights are in the (0,1) domain and must be $\sum_{i=1}^{\#qid}w_{i}^{qid}=1$ and $\sum_{j=1}^{\#SA}w_{j}^{SA}=1$. According to the λ-diversity requirements, if two data records *r*_*a*_ and *r*_*b*_ have lower distance between quasi-identifier attributes and higher distance between the sensitive attributes, means they are closer to each other.

*Pearson distance function*. Eq (6) is the designed Pearson distance function based on the λ-diversity requirements. This function calculates the Pearson distance between numeric records *r*_*a*_ and *r*_*b*_ that have quasi-identifier (QID) and sensitive attributes (SA). Eq (4) is the Pearson similarity function between two data records.

$$PearsonSimilarity(r_{a},r_{b})=\frac{\sum_{k=1}^{m}\left(r_{ak}-\bar{r_{a}}\right).(r_{bk}-\bar{r_{b}})}{\sqrt{\sum_{k=1}^{m}{\left(r_{ak}-\bar{r_{a}}\right)}^{2}}\sqrt{\sum_{k=1}^{m}{\left(r_{bk}-\bar{r_{b}}\right)}^{2}}}$$ (4)

In Eq (4) $\bar{r_{a}}=\frac{1}{m}\sum_{k=1}^{m}r_{ak}$ and $\bar{r_{b}}=\frac{1}{m}\sum_{k=1}^{m}r_{bk}$

In Eq (5) the Pearson distance between two data records is calculated by subtracting the Pearson similarity function from 1.

$$PearsonDistance(r_{a},r_{b})=1-PearsonSimilarity(r_{a},r_{b})$$ (5)

In Eq (6), we have used the Pearson distance function and designed the distance function of two data records *r*_*a*_ and *r*_*b*_ with QID and SA attributes.

$$dist(r_{a},r_{b})=PearsonDistance({r_{}}_{a}^{qid},{r_{}}_{b}^{qid})+(1-PearsonDistance({r_{}}_{a}^{SA},{r_{}}_{b}^{SA}))$$ (6)

According to Eq (4) the Pearson distance function is only usable on datasets with at least two sensitive attributes.

#### 4.2.3 Phase 3: Validation

In the third phase of the proposed computing model, the treeRDD is updated and, then the termination condition is examined. In this phase, first using a mapToPair() transformation per each final seed of seedRDD, a node is appended to the corresponding parent in treeRDD and the tree is updated. Then, using another mapToPair() transformation, the termination condition of the computing model is checked. Therefore, clusters with more than 2*k records are sent to phase 1 for performing the next execution round (for selecting ḱ seeds), and the operations for clusters with less than 2*k records are finished because these clusters can not be divided further. The termination condition of the operations is that the number of data records of all clusters be less than 2*k records. Finally, for generalizing data records of each cluster, the data values of each attribute are replaced with the domain [min, max] of that attribute, and then the data records with generalized values are placed in anonymizedRDD. The operations of the validation phase are according to instruction_set 3.

**Table:** 

Instruction_set *3*. Validation Phase
Input: seedRDD, treeRDD, pairdataRDD Output: updated treeRDD, anonymizedRDD
1. temp2 = treeRDD.join(seedRDD); 2. treeRDD = temp2.mapToPair(…); 3. JavaPairRDD<Key, Value> anonymizedRDD = pairdataRDD.mapToPair(…);

## 5. Evaluation of the proposed computing model

This section first introduces the commonest used datasets in the data anonymization field. Then, the most important criteria for evaluating the distributed anonymization methods are reviewed, and finally, the results of the proposed computing model are presented.

### 5.1 Dataset

In this section, the Poker hand dataset, which has been used in recent researches, is introduced. It is available in the UCI machine learning repository [52]. This dataset is used for classification and contains 1,025,010 data records with 11 numerical attributes. In the recent data anonymization researches, the first 10 attributes have been used as quasi-identifier attributes (QID), and the 11^th^ attribute (the class label) has been used as the sensitive attribute.

### 5.2 Performance evaluation criteria

The most significant performance evaluation criteria of the distributed anonymization methods are examined in the following.

- **Information loss.** Due to the use of the generalization operator in the anonymization process, information loss occurs in published data. Information loss is calculated using Eq (7) which r and s are the number of data records and the number of attributes, respectively. upper_ij_ and lower_ij_ are the upper and lower bounds of the j^th^ attribute in the i^th^ record after the anonymization. max_j_ and min_j_ are the greatest and smallest values of the j^th^ attribute before anonymization.

$$Information\;loss=\frac{1}{r.s}\sum_{i=1}^{r}\sum_{j=1}^{s}\frac{\left|{upper}_{ij}-{lower}_{ij}\right|}{\left|{max}_{j}-{min}_{j}\right|}$$ (7)

- **Classifier evaluation criteria.** The major challenge in data privacy is the trade-off between privacy and data utility. Therefore, to determine how much the anonymization method has negatively affected the classifier evaluation criteria, both the original and the anonymized datasets are classified, and the results of both cases are compared based on accuracy and F1-measure criteria.
- **Running time.** The running time of the implemented algorithms is measured in milliseconds (ms). The runtime severely depends on the platform computing capabilities and the time complexity of the algorithms. Runtime is used to prove the speed and scalability of the designed methods.

### 5.3. Evaluation of computing model

This paper has proposed a distributed computing model for satisfaction of the λ-diversity model concerning enhancing the Information loss, classifier evaluation criteria, and runtime on the numerical datasets. Since according to our research conditions the previous works had a research distance from our research, then Zakerzadeh’s et al. [36], which is a close and state-of-the-art method to our research, was the best option for comparisons. For evaluating the proposed computing model, we have implemented the ḱ-means algorithm for it and have run it with ḱ = 3 (number of seeds), k = 10,60,120,200, and λ = 4,5,6 on the poker hand dataset. Then, we have compared the results with the Zakerzadeh et al. [36] method, which is a distributed λ-diversity-based method on the MapReduce platform. The experimental results have proved the superiority of the proposed computing model.

### • Evaluation of information loss

**Fig 6:**
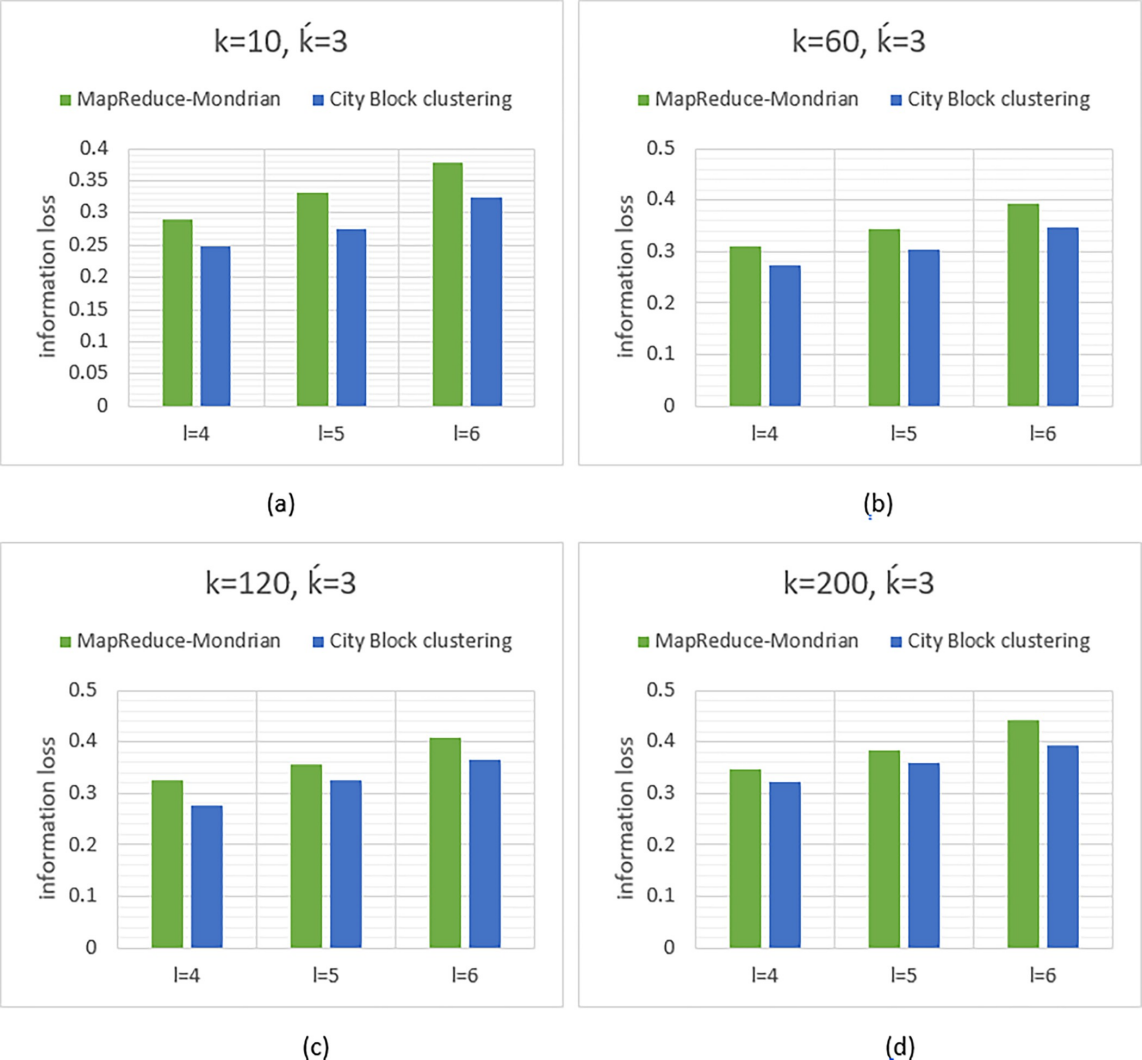
Information loss in anonymous poker hand with ḱ = 3 and different values of k and **λ**.

In Fig 6, the information loss diagrams are shown in the anonymization of the poker hand dataset. Experimental results have shown that the ḱ-means clustering with city block distance function results in lower information loss than the MapReduce-Mondrian method. For example, in Fig 6(A), the obtained values for information loss in MapReduce-Mondrian and city block clustering methods with λ = 4 are 0.291 and 0.247 respectively, which shows lower information loss in the city block clustering method. The diagrams have demonstrated that the higher value of k, the more information loss, and this is due to the growth in the size of the clusters in which the data are arranged. The rest illustrated results in the diagrams of Fig 6 prove that the proposed method leads to less information loss and thus enhances the data utility for analytical methods such as classification algorithms. As a result, a better trade-off is established between privacy and data utility.

### • Evaluation of Accuracy

For evaluating the classifiers, both original and anonymous datasets are classified using the SparkMLlib library. One of the purposes of this paper is to achieve the minimum drop in the classifier evaluation criteria after anonymization (not increasing the accuracy and F1-measure). Therefore decision tree and k-nearest-neighbor classifiers have been used that have acceptable performance on the poker dataset. Since our research aim is not to improve the performance of one classifier hence the classifier type is not very significant for the anonymization process. The chosen classifiers have been applied in the experiments and the evaluation criteria have been obtained according to Table 4. The results of Tables 4, 5 and 6 have been obtained from 5-fold cross-validation.

**Table 4:** Classifier evaluation criteria on the poker hand.

	Decision Tree	k-Nearest Neighbors
**Accuracy**	54.87%	70.45%
**F1-measure**	48.53%	67.82%

**Table 5:** Accuracy criterion on the anonymous poker hand for **λ** = 4, ḱ = 3 and different values of k.

	MapReduce-Mondrian			City block Clustering		
	K = 10	K = 60	K = 120	K = 10	K = 60	K = 120
**Decision Tree**	52.16%	52.01%	51.83%	53.18%	52.71%	52.32%
**k-Nearest Neighbors**	67.82%	67.43%	66.92%	69.12%	68.43%	67.78%

**Table 6:** F1-measure criterion on the anonymous poker hand for **λ** = 4, ḱ = 3 and different values of k.

	MapReduce-Mondrian			City block Clustering		
	K = 10	K = 60	K = 120	K = 10	K = 60	K = 120
**Decision Tree**	46.01%	45.69%	45.42%	47.05%	46.45%	46.15%
**k-Nearest Neighbors**	65.15%	64.42%	63.94%	66.28%	65.58%	65.12%

The poker hand dataset has been anonymized with values of k = 10, 60, 120, and λ = 4, and ḱ = 3. In Table 5, the acquired values of accuracy have been illustrated for the decision tree and K-nearest neighbors classifiers. As shown in Table 5, by increasing k, the value of accuracy decreases, which is due to more information loss according to Eq (7) for bigger clusters. The obtained accuracy with k = 10 for decision tree and k-nearest neighbors classifiers are 53.18% and 69.12% respectively, which in comparison to Table 4 has lower drop than the MapReduce-Mondrian method. Therefore, due to the lower information loss in the proposed method, the obtained accuracy is better than MapReduce-Mondrian and a better trade-off is established between privacy and data utility.

In Table 6, the acquired values of the F1-measure have been illustrated for the decision tree and K-nearest neighbors classifiers. As shown in Table 6, by increasing k, the value of accuracy decreases, which is due to more information loss according to Eq (7) for bigger clusters. The obtained F1-measure with k = 10 for decision tree and k-nearest neighbors classifiers are 47.05% and 66.28% respectively, which in comparison to Table 4 has lower drop than the MapReduce-Mondrian method. Therefore, due to the lower information loss in the proposed method, the obtained F1-measure is better than MapReduce-Mondrian and a better trade-off is established between privacy and data utility.

### • Evaluation of running time

**Fig 7:**
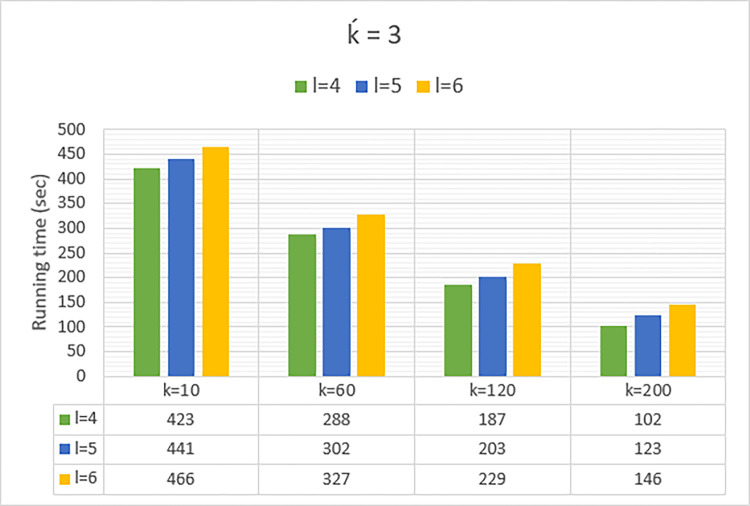
Runtime (sec) of the city block clustering on poker Hand dataset for ḱ = 3.

In Fig 7, the runtime of the anonymization process on the poker hand dataset is shown. We have conducted the experiments on a virtual cluster with a 3.40GHz Intel Core i7 processor, and 16GB of memory. The implementations have been accomplished with Java and Apache Spark libraries in the Eclipse IDE. Given that Spark-based applications are 100 times faster in-memory than MapReduce-based applications, it is not fair to compare their runtime, and in this section, only the runtime of Spark is reported. The results of Table 6, have been measured on second. The runtime is reduced with raising k because fewer execution rounds have to be executed for forming larger clusters. On the other hand, the runtime is increased with raising λ, because more operations are required for forming λ-diverse clusters. Therefore, runtime results prove the scalability of the proposed computing model. By improving the computational capacity and the number of worker nodes, anonymizing the large-scale datasets is more convenient.

## 6. Conclusion

The rapid growth of data generation and producing large-scale datasets has incurred great challenges to traditional data anonymization tools. Apache Spark is the state-of-the-art framework for applications that have iterative operations or real-time statistical analysis on large-scale datasets. At the same time, Due to the nature of the Sparks, it is used to develop scalable and high-speed applications. For this reason, we have applied Apache Spark for big data anonymization and have presented a guideline on this topic. First, we have introduced the Spark cluster architecture, ecosystem, programming model, and processing mechanism. Then, we surveyed the use of Spark-based applications in the big data anonymization domain by using partition-based data clustering algorithms. In this context, two distance functions have been designed for preserving the λ-diversity privacy model requirements using City block and Pearson functions. All implementations are designed by Transformations and Actions on RDDs. Therefore, the experimental results have illustrated that this computing model established a better trade-off between privacy and data utility. By this working model, and executing parallelized in-memory operations on the executor of worker nodes, Spark can address challenges related to runtime, scalability, and performance. This proposed computing model is efficient, flexible, and with a little change can be adapted to preserve all privacy models. Our study means that researchers who wish to become involved in this field can now acquire a general knowledge of the use of Spark in big data anonymization.

## Supporting information

S1 Data (XLSX)
